# Premature Burial

**Published:** 1910-03-05

**Authors:** 


### The Hospital

A Journal of

The Medical Sciences and Hospital Administration.

New Series. No. 159, Vol. VI. [No. 1231, Vol. XLVII.] SATURDAY, MARCH 5, 1910.

### Premature Burial.

From the earliest times the fear of premature burial has been felt by many, and curious and strange methods have been adopted to prevent the possibility of individuals being consigned to their graves before life was extinct. Tradition records many cases where, in spite of these precautions, such unfortunate accidents have happened. It may be that tradition is an uncertain and erring guide. And yet underlying all tradition, as Dieulafoy said, is a solid substratum of truth which the thoughtful investigator must take into consideration. The tale of the Cologne goldsmith's wife, that survives in the legend of the neighing horses, may be weird, bizarre, and, from a scientific point of view, demonstrably ludicrous, but its germ is to be found in the recorded fact that in times of epidemics, when the dying were huddled away with the dead, mistakes did occur, and one or two such were rectified by the unexpected resurrection of the "dead." In cases where burial took place in commodious family vaults the changes in the position of the coffin, produced by atmospheric and other physical factors and startlingly disclosed when the vaults were opened to receive new bodies, doubtless gave an impetus to the belief in the comparative frequency of such mistakes. There can be little doubt that a patient and thorough investigator could light on apparently authenticated instances which would strongly support the fears of the nervous. The public, however, is apt to pay more attention to exceptions than to the rule that they proverbially prove. For that reason it is probable that the efforts of the well-meaning Association for the Prevention of Premature Burial cause a great deal of unnecessary anguish of mind to many sensitive persons, whose imaginations are apt to run riot when they hear one of these horrible stories that the morbid-minded Wiertz has so strikingly portrayed in his peep-hole canvas, "The Buried Alive." Who looks at this awful picture, so vivid in its colouring, so fine in its technical accuracy of detail, without a shudder and a passing fear that such things may, after all, happen in our civilised days? The medical man remembers that on occasions he has found it difficult, without applying some of the common and finer tests, to certify death in a patient dying of a lingering disease, but his knowledge forbids him believing that such difficulties as he may have experienced in his own practice can ever have caused his fellow practitioner to make so gruesome a mistake in a similar case. The public has no such knowledge; it relies on the exceptional cases, and glibly credits the statement—true enough in a limited sense—that there is no certain proof of death. While it is certainly true that no single sign can be absolutely relied upon to prove that life is extinct, all practitioners will agree that several signs taken in combination and methodically applied are sufficiently accurate to obviate the possibility of mistake. That mistakes are made we cannot, unfortunately, dispute. The evidence given at the annual meetings of the Association already alluded to keep these mischances constantly before the public and professional eye, and it would be futile to deny that much of this evidence is unimpeachable. But while we admit this much, we should be sorry to accept the case presented by the Association in its entirety, so far, at least, as this country is concerned, for to do so would be to admit that the medical profession is unworthy of the confidence which the public places in it. Sifting the mass of evidence already presented, it is clear that some of the cases are capable of an interpretation widely different from that which the timorous attach to them, and, on the other hand, it is equally clear that there is a tendency to exaggerate dangers the existence of which no one presumes to deny. Much has been made of the cataleptic condition and the probability of mistaking it for death, which has formed the basis of one of Poe's weird narratives. As a matter of fact catalepsy, of such a nature as to be liable to be confounded with total exitus, is extremely rare—so rare that we doubt if any practitioner with a large experienco ef nervous conditions has met with more than one or two instances. Further, even in such extremely rare conditions the usual tests are applicable, and to the trained medical man at least clearly prove the nature of the case. The stethoscope and the mirror held before the patient's mouth are usually sufficient to demonstrate that the patient is alive, and we should want much more conclusive evidence than such as has been brought forward up to the present to feel convinced that cataleptic patients have been consigned to their coffins before life was totally extinct.

Nevertheless, the Association for the Prevention of Premature Burial has done yeoman service in drawing attention to the glaring defects in our system of death certification. Under present conditions the regulations for making out such certificates are exceedingly lax, and ought to be amended as soon as possible. That qualifying sentence " as I am informed " should not be allowed to appear on a document of such primary importance. The law permits a medical man to certify death on the mere assumption that a lay witness is capable of diagnosing death. In most cases the medical attendant, from his knowledge of the case, is absolutely justified in signing such a certificate, but it is only fair to assume that in some cases such justification is not absolute, and that personal inspection of the body, personal verification of death, is imperatively essential. There are occasions in general practice when a medical man is scarcely warranted in giving an ordinary certificate. A well-to-do patient, let us say, has fallen down in the street and been carried home, and the medical attendant on being called in finds him dead. That person may have suffered from heart disease or from some other lesion which could be held accountable for his sudden death, and most medical men, with a laudable desire to save the friends the worry and publicity of a coroner's inquest, would not hesitate for a moment to certify that death was due to natural causes. Similarly, a patient may suddenly become violently ill, with vomiting and collapse; there is a history, only too easily to be got by leading questions, of something unwholesome having been eaten; the diagnosis of ptomaine poisoning or acute gastritis is made, and the patient's death is certified accordingly. Yet it is within the realm of probability that in a small percentage an autopsy might have revealed another cause of death in both these cases. We have no wish to labour this argument or to add to the fears of the nervous public, but we are primarily concerned with medical men, and general practitioners know quite well that it is no overstatement of the matter to allege that the present system of death certification is not an absolute safeguard against the dangers which an adequate and efficient system ought to prevent.

